# Tsukushi is essential for the development of the inner ear

**DOI:** 10.1186/s13041-020-00570-z

**Published:** 2020-03-03

**Authors:** Toru Miwa, Kunimasa Ohta, Naofumi Ito, Satoko Hattori, Tsuyoshi Miyakawa, Toru Takeo, Naomi Nakagata, Wen-Jie Song, Ryosei Minoda

**Affiliations:** 1 Department of Otolaryngology-Head and Neck Surgery, Kitano Hospital, Tazuke Kofukai Medical Research Institute, 2-4-20 Ougimaci, Kita-ku, Osaka, 5308084 Japan; 2 Department of Otolaryngology-Head and Neck Surgery, Graduate School of Medicine, Kyoto University, 54 Shogoin Kawahara-cho, Sakyo-ku, Kyoto, 6068507 Japan; 3 Departments of Otolaryngology-Head and Neck Surgery, Graduate School of Medicine, Kumamoto University, 1-1-1 Honjo, Kumamoto, 8608556 Japan; 4 Otolaryngology-Head and Neck Surgery, JCHO Kumamoto General Hospital, 10-10 Toricho, Yatsushiro, 8668660 Japan; 5 Department of Developmental Neurobiology, Graduate School of Life Sciences, Kumamoto University, 1-1-1 Honjo, Kumamoto, 8608556 Japan; 6 Program for Leading Graduate Schools HIGO Program, Kumamoto University, 2-2-1 Honjo, Kumamoto, 8608556 Japan; 7 Global COE Cell Fate Regulation Research and Education Unit, Kumamoto University, 2-2-1 Honjo, Kumamoto, 8600881 Japan; 8 Japan Agency for Medical Research and Development (AMED), Tokyo, 1000004 Japan; 9 Division of Systems Medical Science, Institute for Comprehensive Medical Science, Fujita Health University, 1-98 Dengakugakubo, Kutsukak, Toyoake, 4701192 Japan; 10 Division of Reproductive Engineering, Center for Animal Resources and Development (CARD), Kumamoto University, 2-2-1 Honjo, Kumamoto, 8600881 Japan; 11 Department of Sensory and Cognitive Physiology, Graduate School of Life Sciences, Kumamoto University, 1-1-1 Honjo, Kumamoto, 8608556 Japan

**Keywords:** Tsukushi, Hair cell stereocilia, Sox2, BMP4, Hearing

## Abstract

Tsukushi (TSK)—a small, secreted, leucine-rich-repeat proteoglycan—interacts with and regulates essential cellular signaling cascades. However, its functions in the mouse inner ear are unknown. In this study, measurement of auditory brainstem responses, fluorescence microscopy, and scanning electron microscopy revealed that TSK deficiency in mice resulted in the formation of abnormal stereocilia in the inner hair cells and hearing loss but not in the loss of these cells. TSK accumulated in nonprosensory regions during early embryonic stages and in both nonprosensory and prosensory regions in late embryonic stages. In adult mice, TSK was localized in the organ of Corti, spiral ganglion cells, and the stria vascularis. Moreover, loss of TSK caused dynamic changes in the expression of key genes that drive the differentiation of the inner hair cells in prosensory regions. Finally, our results revealed that TSK interacted with Sox2 and BMP4 to control stereocilia formation in the inner hair cells. Hence, TSK appears to be an essential component of the molecular pathways that regulate inner ear development.

## Introduction

Tsukushi (TSK) is a small, secreted leucine-rich-repeat proteoglycan and extracellular signaling molecule that participates in various developmental processes in vertebrates [1–3]. The diverse functions of TSK depend on its ability to bind and interact with various intermediate molecules of some major signaling pathways. TSK interactions with BMP, FGF, TGF-β, and Wnt signaling pathways have been previously reported [1, 4–6] and shown to affect the development of the central nervous networks [7, 8], as well as wound healing [9]. TSK is reportedly expressed in the chick retina [3, 10], the subventricular zone and hippocampus in the mouse brain [8], and dermal hair follicles in mice [9]. However, TSK function and localization in the inner ear have not yet been investigated. Indeed, the molecular mechanisms underlying the inner ear development and acquisition of normal hearing are still unknown [11].

Accordingly, we studied TSK expression and function during inner ear morphogenesis and after birth. In addition, we investigated TSK interaction with BMP. BMP signaling is crucial for the development of the inner ear, and its gradient expression in the developing inner ear causes dysmorphogenesis of the inner ear [12, 13]. Therefore, after confirming TSK expression, we performed histological examination to investigate the phenotypes related to TSK deficiency, namely, hearing and changes in cell morphology. We then investigated the molecular mechanisms of TSK interaction with BMP4 by immunostaining and assessing mRNA expression levels. Finally, we investigated specific effects of TSK deficiency on stereocilia of the hair cells (HCs), while leaving the rest of the inner ear morphologically intact.

## Results

### TSK was differentially expressed in various tissues during embryonic development

**Fig. 1:**
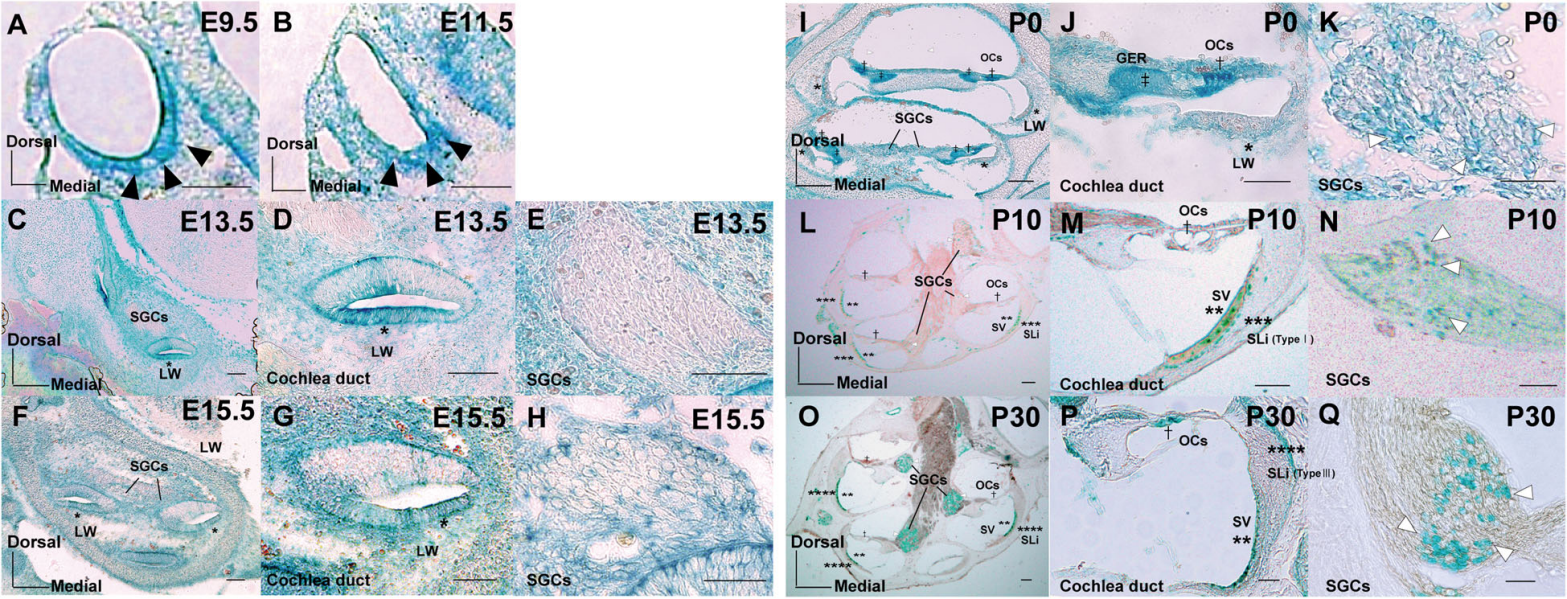
Spatiotemporal expression of TSK in the inner ear. **a**, **b** At E9.5 and E11.5, TSK-expressing cells were localized in the ventral tissues of the otocyst, which would differentiate into the cochlear duct (black arrowheads). **c**-**h** At E13.5 and E15.5, TSK-expressing cells were localized at the LW of the cochlear duct, which would differentiate into SV (asterisks in **c**, **d**, **f**, **g**), and not SGCs (**e**, **h**). **i**-**k** At P0, TSK was expressed in some SGCs (white arrowheads in **i**, **k**,), the OC (dagger in **i**, **j**), LW, which differentiates into the SV and SLi (asterisks in **i**, **j**), and in the GER, which differentiates into the spiral limbus and tectorial membrane (double dagger in **i**, **j**). **l**-**n** At P10, TSK was expressed in some SGCs (white arrowheads in **l**, **n**), the OC (dagger in **l**, **m**), the SV (double asterisk in **l**, **m**), and type I fibrocytes of the SLi (triple asterisk in **l**, **m**). (**o**-**q**) At P30, TSK was expressed in some SGCs (white arrowheads in **o**, **q**), the OC (dagger in **o**, **p**), the SV (double asterisk in **o**, **p**), and type III fibrocytes of the SLi (quattro asterisk in **o**, **p**). GER: greater epithelial ridge, LW: lateral wall, OC: organ of Corti, SGC: spiral ganglion cell, SV: stria vascularis, SLi: spiral ligament. Scale bars represent 100 μm (**a** and **b**) or 50 μm (**c**-**q**)

For the first time, we mapped TSK localization in the inner ear. To do this, embryos, in which the *LacZ* reporter gene was introduced within the coding exon of *TSK*, were fixed, cryosectioned, and stained with X-gal. At embryonic day 9.5 (E9.5) and E11.5, TSK-expressing cells were localized in the ventral otocyst tissues, which eventually differentiate into the cochlear duct (Fig. 1a, b). At E13.5 and E15.5, TSK-expressing cells were detected at the lateral wall of the cochlear duct, which would later differentiate into the stria vascularis and spiral ligament, and not to spiral ganglion cells (SGCs) (Fig. 1c-h). At postnatal stage 0 (P0), TSK-expressing cells were observed in some SGCs, the OC, inside and outside of the lateral wall that differentiates into the stria vascularis and spiral ligaments, and the greater epithelial ridge, which differentiates into the spiral limbus and tectorial membrane (Fig. 1i-k). At P10, TSK was expressed in some SGCs, the OC, the stria vascularis, and type I fibrocytes in spiral ligaments (Fig. 1l-n). Finally, at P30, TSK was localized in SGCs, the OC, the stria vascularis, and type III fibrocytes in spiral ligaments (Fig. 1o-q).

### TSK deficiency caused hearing abnormalities

**Fig. 2:**
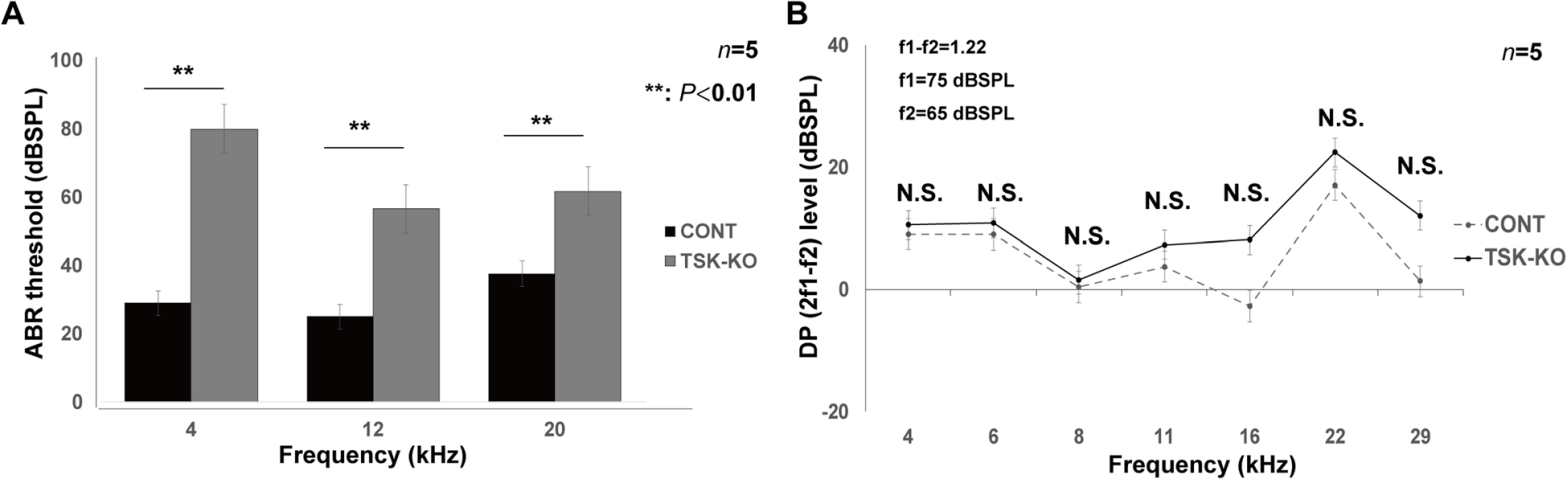
Loss of TSK signaling in the cochlea caused hearing loss. **a** Hearing thresholds in the CONT and the TSK-KO mice at P30. Differences were significant at all frequencies. **, *p* = 0.002 at 4 kHz; **, *p* = 0.002 at 12 kHz; **, *p* = 0.004 at 20 kHz. **b** DPOAE levels in the CONT or the TSK-KO mice at P30. Differences were not statistically significant at all tested frequencies. *p* = 0.29 at 4 kHz, *p* = 0.27 at 5.6 kHz, *p* = 0.38 at 8 kHz, *p* = 0.29 at 11.3 kHz, *p* = 0.06 at 16 kHz, *p* = 0.19 at 22.6 kHz, *p =* 0.06 at 29 kHz. ABR: auditory brainstem response, N.S.: not significant

At P30, frequency-averaged auditory brainstem response (ABR) thresholds for control (CONT) and TSK-knock out (KO) mice were 29.0 ± 5.83 and 80.0 ± 9.29 dBSPL at 4 kHz, 25.0 ± 9.48 and 56.5 ± 15.2 dBSPL at 12 kHz, and 37.6 ± 4.36 and 61.7 ± 14.3 dBSPL at 20 kHz, respectively. Differences were significant at all frequencies (Fig. 2a; *p* = 0.002 at 4 kHz, *p* = 0.002 at 12 kHz, and *p* = 0.004 at 20 kHz). On the other hand, levels of the frequency-averaged distortion product of otoacoustic emission (DPOAE) were 9.08 ± 3.34 and 10.5 ± 6.4 dBSPL at 4 kHz, 8.99 ± 7.63 and 10.8 ± 4.22 dBSPL at 5.6 kHz, 0.38 ± 8.96 and 1.6 ± 7.66 dBSPL at 8 kHz, 3.77 ± 12.9 and 7.34 ± 13.9 dBSPL at 11.3 kHz, − 2.70 ± 15.7 and 8.11 ± 11.1 dBSPL at 16 kHz, 17.1 ± 13.6 and 22.4 ± 10.7 dBSPL at 22.6 kHz, and 1.35 ± 9.99 and 12.1 ± 12.4 dBSPL at 29 kHz, respectively. These differences were not statistically significant at all tested frequencies (Fig. 2b; *p* = 0.29 at 4 kHz, *p* = 0.27 at 5.6 kHz, *p* = 0.38 at 8 kHz, *p* = 0.29 at 11.3 kHz, *p* = 0.06 at 16 kHz, *p* = 0.19 at 22.6 kHz, and *p* = 0.06 at 29 kHz).

Cognitive function was evaluated using the fear-conditioning test to clarify the hearing loss and to examine learning and memory function in the TSK-KO mice. In this test, freezing responses between the CONT and TSK-KO mice in the conditioning phase (auditory stimulus and foot shock, 2-min interstimulus interval) or contextual testing (one day after conditioning for 300 s) were not significantly different (Additional file 1, conditioning phase, *p* = 0.94; contextual testing, *p* = 0.75). Levels of freezing in the TSK-KO mice significantly decreased during the auditory cue in an altered context (auditory stimulus for 180 s) compared with that in the CONT mice (Additional file 1, *p* = 0.02).

### TSK deficiency caused the formation of abnormal stereocilia, but not loss of hair cells

**Fig. 3:**
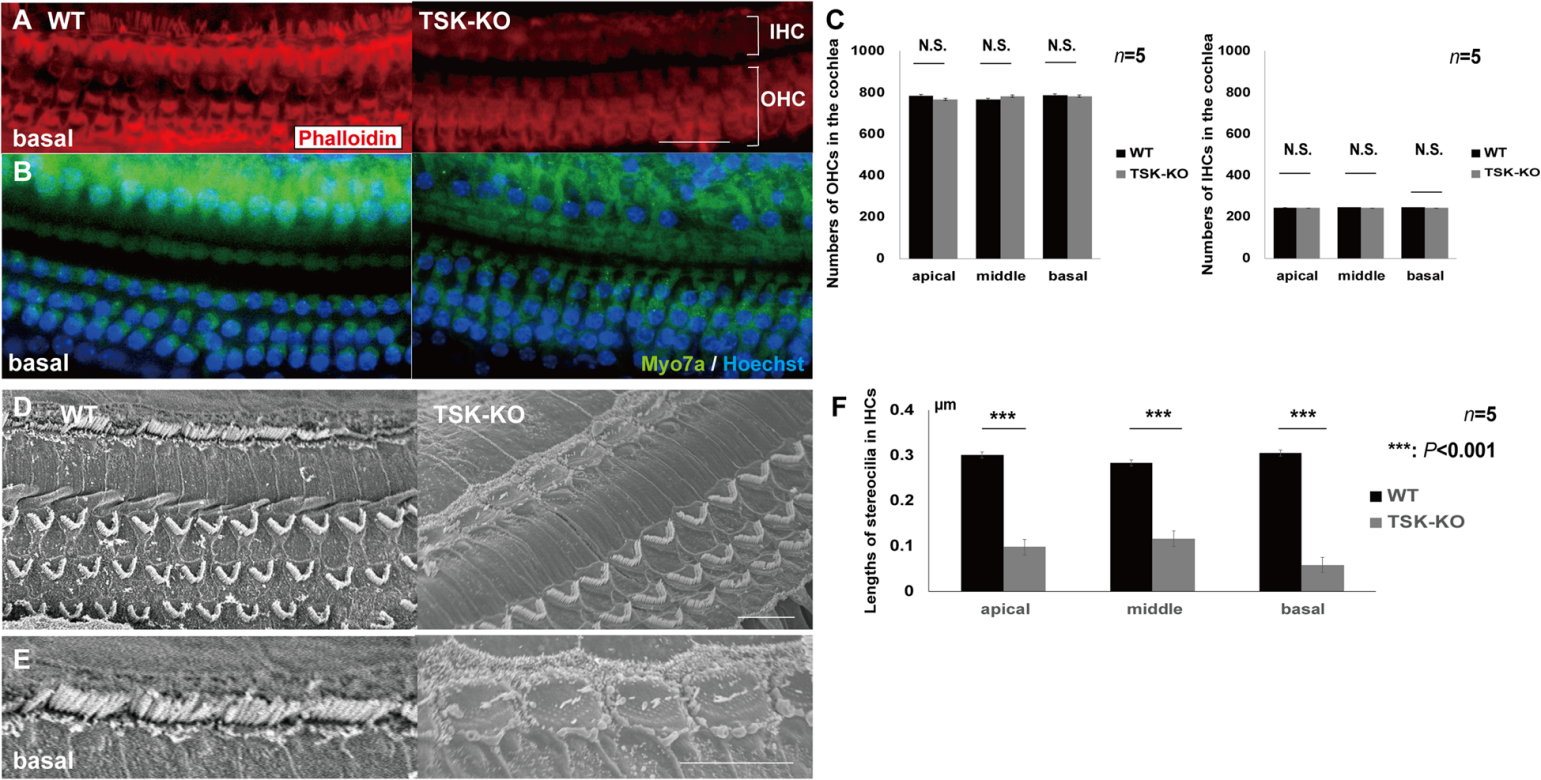
Enumeration of the hair cells in surface preparations of cochleae at P30. **a**, **b** Phalloidin staining and immunostaining with anti-Myo7a of the hair cells at basal turns in the CONT (left) and TSK-KO mice (right). **c** The numbers of outer and inner hair cells in the CONT and TSK-KO mice were not significantly different; *p* = 0.09, 0.30, and 0.18, respectively, for apical, middle, and basal outer hair cells; *p* = 0.46, 0.29, and 0.45, respectively, for apical, middle, and basal inner hair cells. **d**, **e** Scanning electron micrographs of the hair cells in the CONT and TSK-KO mice. **f** Length of stereocilia in the inner hair cells in the CONT and TSK-KO mice. Stereocilia in the inner hair cells were significantly shortened and dislocated in the TSK-KO mice. ****, *p* < 0.0001 at all turns. IHC: inner hair cell, OHC: outer hair cell, N.S.: not significant, WT: wild-type

Morphology of the HCs, revealed by Phalloidin staining and anti-Myo7a staining, appeared normal in the CONT and TSK-KO mice (Fig. 3a, b). For the quantitative analysis of the body of hair cells, we counted the number of HCs. The numbers of the HCs at apical, middle, and basal turns were not significantly different (Fig. 3c) with *p* = 0.09, 0.30, and 0.18, respectively, for the outer HCs, and *p* = 0.46, 0.29, and 0.45, respectively, for the inner HCs. However, stereocilia in the inner HCs were significantly shortened and dislocated in the TSK-KO mice (Fig. 3d-f, *p* < 0.0001 for all turns), as visualized by scanning electron microscopy. Stereocilia in the outer HCs were unaffected. Stereocilia of inner HCs at P0 and P6 were likely to be shortened in the surface preparation; however, a quantitative analysis could not be performed from our results (Additional file 2). As HC development has been shown to be related to the expression and localization of Sox2, which is known to be regulated in other pathways by BMP4 via TSK, we next investigated the expression and localization of all these proteins at different developmental stages in the mouse.

### TSK loss suppressed Sox2 and redistributed BMP4

**Fig. 4:**
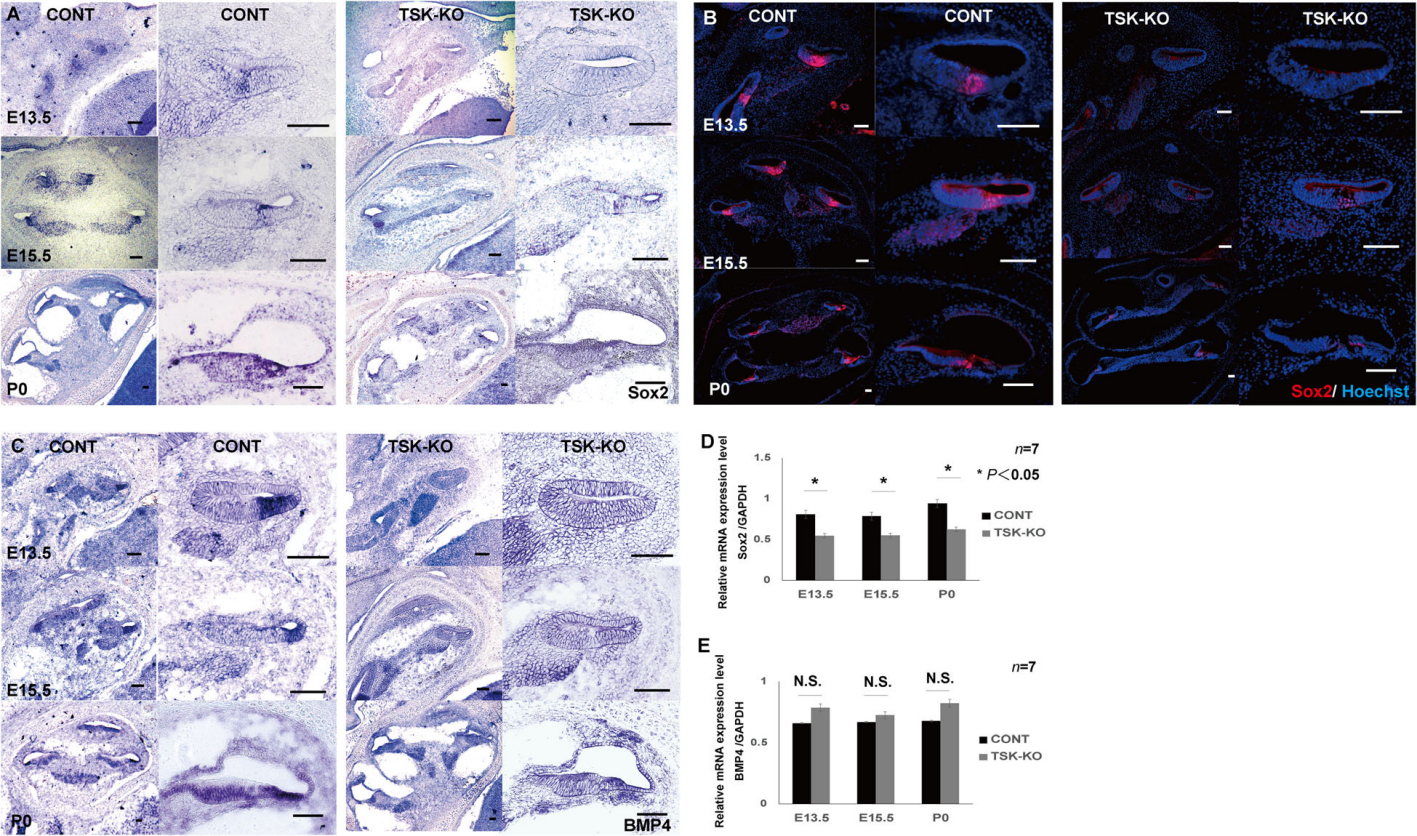
Dynamic cochlear gene expression in the CONT and TSK-KO mice. **a**, **b** Sox2 was detected in prosensory regions by in situ hybridization and immunolabeling in the CONT mice but was diminished in the TSK-KO mice at E13.5, E15.5, and P0. **c** BMP4 was detected by in situ hybridization in nonprosensory regions and outer sulcus in the CONT. In the TSK-KO, BMP4 was diminished in the outer sulcus, but sparsely distributed around the cochlear epithelium (**d**) qRT-PCR for Sox2 mRNA in the cochlear epithelium revealed that Sox2 mRNA was significantly diminished in TSK-KO as compared to its level in CONT. *, *p* = 0.03 at E13.5; *, *p* = 0.04 at E15.5; *, *p* = 0.01 at P0. **e** qRT-PCR for BMP4 RNA in the cochlear epithelial tissue revealed that BMP4 mRNA increased in the TSK-KO, but only marginally. *p* = 0.23 at E13.5; *p* = 0.37 at E15.5; *p* = 0.19 at P0. N.S.: not significant

We examined Sox2 and BMP4 expression in the cochlea at various developmental stages in mice to assess differentiation of the HCs. Sox2 was detected by in situ hybridization and immunolabeling in prosensory regions in the CONT mice but was diminished in the TSK-KO mice at E13.5, E15.5, and P0 (Fig. 4a, b). Quantitative reverse-transcription polymerase chain reaction (qRT-PCR) of the cochlear epithelial tissue revealed that Sox2 mRNA was also significantly diminished in the absence of TSK (Fig. 4d), with *p* = 0.03, 0.04, and 0.01 at E13.5, E15.5, and P0, respectively, as compared to CONT tissues. Similarly, BMP4 was detected by in situ hybridization in nonprosensory regions and outer sulcus in the CONT mice. In the TSK-KO mice, BMP4 was diminished in the outer sulcus but thinly distributed around the cochlear epithelium (Fig. 4c). Finally, qRT-PCR of the cochlear epithelial tissue revealed that BMP4 mRNA increased in the TSK-KO, but only marginally (Fig. 4e), with *p* = 0.23, 0.37, and 0.19 at E13.5, E15.5, and P0, respectively. Considering these TSK-dependent, region-specific changes in Sox2 and BMP4 expression, we next investigated changes in the cellular composition of the tissue.

### Spiral ganglion cells were diminished at the cochlear basal turn in the TSK-KO mice

**Fig. 5:**
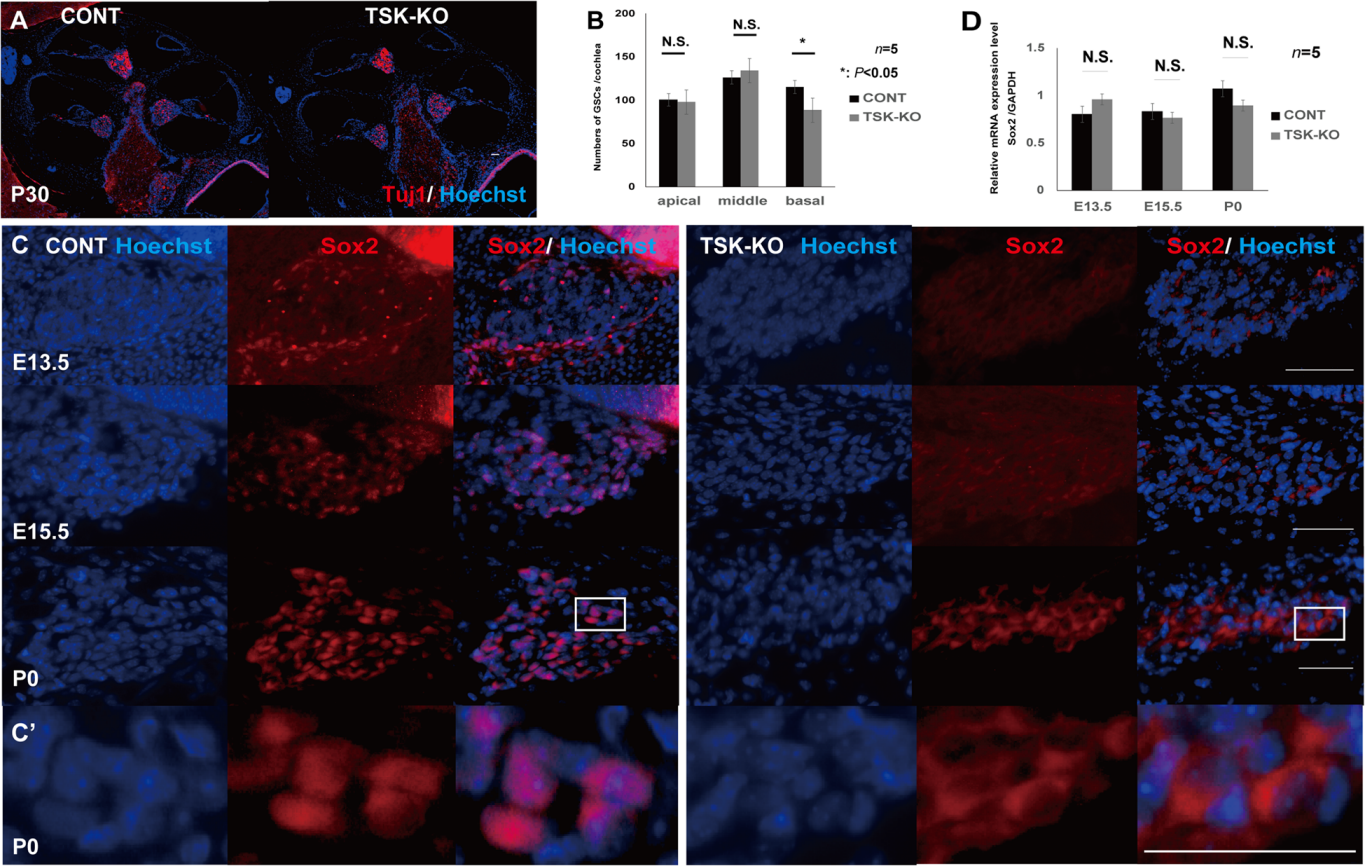
Assessment of spiral ganglion cells in the CONT and TSK-KO mice. **a** Gross morphology. **b** Differences in numbers were not significant at apical (*p* = 0.39) and middle (*p* = 0.15) turns, but were significant at basal turns (*, *p* = 0.02). **c** Immunolabeling showed that Sox2 accumulated in the nuclei of the spiral ganglion cells in the CONT mice but was suppressed in the TSK-KO mice at E13.5, E15.5, and P0. Sox2 translocated to the cytosol at P0 in the TSK-KO mice. (C′) Magnified view of the white hollow square in C. **d** qRT-PCR for Sox2 mRNA in the spiral ganglion tissue collected by laser microdissection did not detect significant differences in Sox2 mRNA between the CONT and TSK-KO mice. *p* = 0.09 at E13.5; *p* = 0.34 at E15.5; *p* = 0.21 at P0. N.S.: not significant

The average number of the SGCs in the CONT and TSK-KO mice was 100.6 ± 10.1 and 98.0 ± 10.1 at the apical turn, 126.4 ± 12.8 and 134.3 ± 24.9 at the middle turn, and 115.5 ± 20.4 and 88.6 ± 25.2 at the basal turn, respectively. Differences were significant at the basal turn (Fig. 5a, b) with *p* = 0.39, 0.15, and 0.02 for the apical, middle, and basal turn, respectively.

### TSK deficiency translocated Sox2 in the spiral ganglion cells

Immunolabeling showed that Sox2 accumulated in the nuclei of the SGCs in the CONT mice but was suppressed in the TSK-KO mice at E13.5, E15.5, and P0 (Fig. 5c). Interestingly, Sox2 translocated to the cytosol at P0 in the TSK-KO mice (Fig. 5c) only at the basal turn. In addition, qRT-PCR did not detect significant differences in Sox2 mRNA between the CONT and TSK-KO mice (Fig. 5d), with *p* = 0.09, 0.34, and 0.21 at E13.5, E15.5, and P0, respectively.

### TSK loss did not affect the stria vascularis, spiral ligament, and endocochlear potentials

**Fig. 6:**
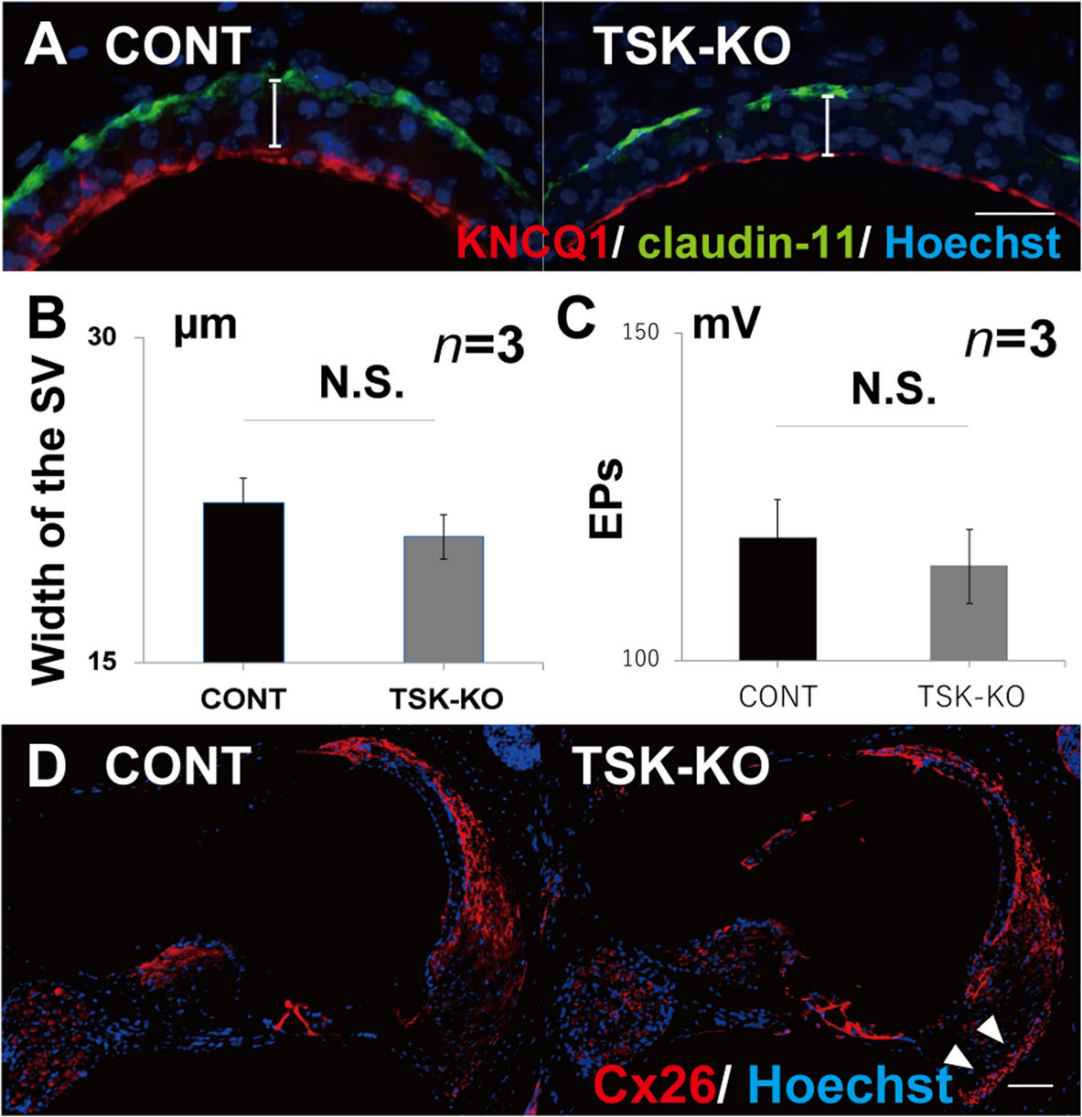
Assessment of the stria vascularis in the CONT and TSK-KO mice. **a**, **b** Thickness of the stria vascularis (SV) (*p* = 0.13) and expression of claudin-11 and the potassium channel KCNQ1, which are markers of the marginal and basal cells, did not differ between the CONT and TSK-KO mice. **c** Endocochlear potentials (EPs) decreased in the TSK-KO mice, but only marginally (*p* = 0.20). **d** In addition, expression of connexion-26 (Cx26), a marker of the spiral ligament, was comparable between the CONT and TSK-KO mice except for the subcentral region below the spiral prominence. N.S.: not significant

Thickness of the stria vascularis (Fig. 6a, b, *p* = 0.13) and expression of claudin-11 and the potassium channel KCNQ1, which are markers of the marginal and basal cells, respectively, did not differ between the CONT and TSK-KO mice. Endocochlear potentials decreased in the TSK-KO mice, but only marginally (Fig. 6c, *p* = 0.20). In addition, expression of connexion-26 (Cx26), a marker of the spiral ligament, was comparable between the CONT and TSK-KO mice except for the subcentral region below the spiral prominence (Fig. 6d).

## Discussion

The molecular mechanisms underlying cochlear development to achieve normal hearing have been challenging to elucidate [11]. Since TSK was previously shown as an organizer inducer that inhibits BMP [3], we investigated its roles in the differentiation of HCs, SGCs, and the stria vascularis. Our results suggested that the main pathomechanism of hearing loss in TSK-KO mice was the shortening of stereocilia of inner HCs, caused by BMP gradient expression due to TSK deficiency (Additional file 3).

### TSK is required for hearing and stereocilia generation in the inner hair cells

We detected TSK in the prosensory and nonprosensory regions of the cochlear epithelial cells of embryonic and adult mice. Further, we found that TSK deficiency in the developing embryonic inner ear redistributed BMP4 to a thin layer throughout the cochlear epithelium, resulting in BMP4 depletion from the outer sulcus and slight accumulation of BMP4 in prosensory regions. Thus, TSK deficiency appears to dispel an inherently asymmetric BMP signaling gradient, which is reportedly critical to inducing and patterning sensory domains in the mammalian cochlea [13]. In addition, loss of BMP signaling in the developing inner ear reportedly causes marked but “incomplete” loss of HCs [12]. We observed that stereocilia in the inner HCs were shortened in the TSK-KO mice, with no loss of the inner or outer HCs. These observations may explain why TSK-KO mice suffer hearing loss as young adults, without loss of the outer HCs.

Moreover, TSK loss suppressed Sox2 at the prosensory regions that differentiate into the OC. Sox2 upregulates the transcription factor Atoh1 in both embryonic and postnatal cochlear progenitor cells, thus triggering differentiation of the HCs [14–17]. Indeed, the inner HCs are markedly susceptible to Atoh1, which controls the formation and organization of the stereocilia in a dose-dependent manner [18, 19]. Further, a stable and precise level of Atoh1 is needed for the development and maintenance of different HCs [20], possibly through the regulation of many essential downstream genes [21]. Previous studies also suggested that the level and duration of Sox2, FGF8, or miR-96 expression impact stereocilia formation, neuronal segregation, and the final HC phenotype, including their number and size [18, 22]. For example, Sox2 deletion at later stages of the embryonic inner ear development elicits BMP4 expression adjacent to the greater epithelial ridge instead of lateral to the OC, as well as the formation of ectopic inner HCs at the boundary of the greater epithelial ridge and the BMP4-expressing tissue [23]. As a corollary, BMP is known to repress Sox2 post-transcriptionally, as was observed during cardiac development [24].

Therefore, we believe that redistribution of BMP4 to the entire cochlear epithelium and Sox2 loss from prosensory regions cause abnormal development of the sensory domain in the cochlea, specifically shortening of the stereocilia in the inner HCs. However, our present study has not described how early changes in BMP4 or Sox2 are related to the P30 phenotypes; therefore, further research is necessary for the changes during postnatal development.

### TSK signaling is required to generate spiral ganglion cells

Primary auditory neurons, also known as spiral ganglion neurons, receive chemical signals from the cochlear HCs and transmit information to the central cochlear nucleus in the brainstem [25]. These primary auditory neurons differentiate from neuronal precursors that delaminate from the otocyst early during the inner ear development [26]. A number of transcription factors, including Neurog1 [27], NeuroD1 [28], GATA3 [29], and Sox2 [16, 30], have been identified to drive neurogenesis and specification of the primary auditory neurons. Expansion and maintenance of neuronal precursors depend on Sox2 [23, 31]. Accordingly, Sox2 expression is downregulated early during embryonic development to allow neurogenesis via Neurog1 and NeuroD1 [32] but is then upregulated in the primary auditory neurons around birth. Our data show that in the absence of TSK, Sox2 is translocated from the nucleus to the cytoplasm at later embryonic stages only at the basal turn. Consequently, fewer SGCs were observed at the basal turn in the adult TSK-KO than in the CONT mice at P30. As the development of the SGCs is known to occur from the basal to the apical turn, we believe that TSK accumulates first in the basal turn as well, followed by Sox2 expression during later embryonic stages. Therefore, Sox2 deficiency at the basal turn due to loss of TSK may explain the decrease in the number of SGCs. Sox2 expression was decreased in the cochlear epithelium of TSK-KO mice due to BMP4 redistribution under TSK deficiency, whereas it was not decreased in SGCs but translated to the cytosol. We speculated that TSK deficiency directly affected Sox2. However, further research is needed to establish whether temporal sweep in the change of localization of Sox2 to the developing SGCs is affected in the mutant.

### TSK signaling is not required to generate endocochlear potentials

In the lateral wall, TSK expression dramatically redistributes from primarily the stria vascularis and type I fibrocytes of the spiral ligament to the stria vascularis and to type III fibrocytes of the spiral ligament. TSK deficiency causes Cx26 expression in type III fibrocytes of the spiral ligament. However, our data do not link TSK deficiency to changes in the morphology of the stria vascularis and spiral ligament, nor to endocochlear potentials. Hence, further research is necessary to clarify these aspects.

### TSK deficiency caused hearing loss but did not impair learning and memory

The results of cochlear morphology assessment, increased ABR at all frequencies, normal DPOAE, and reduced number of SGCs at the basal turn in the TSK-KO mice suggested that shortening of stereocilia of inner HCs was the main pathomechanism of hearing loss in TSK-KO mice. However, there was a possibility of central hearing loss due to cognitive dysfunction. Therefore, we investigated cognitive function using the fear-conditioning test to clarify hearing loss and to examine learning and memory function in the TSK-KO mice. Our results suggest that TSK-KO mice present with hearing loss but do not suffer any impairment of learning and memory function.

The main limitations of our study are as follows. First, we could not explain whether early changes in BMP4 or Sox2 were related to the P30 phenotypes. Second, the relation between TSK deficiency and morphological changes in the stria vascularis and spiral ligament, could not be clarified. Third, we could not establish whether temporal sweep in the change of localization of Sox2 to the developing SGCs was related to TSK deficiency. Further studies addressing these limitations are recommended.

## Materials and methods

### TSK-knockout (KO) mice

TSK-KO C57BL/6 J mice (*Mus musculus domesticus*) were generated by inserting a LacZ/Neo cassette into the TSK-coding exon, as described previously [8]. Wild-type littermates by genotyping were used as CONT. Heterozygous littermates by genotyping were used for LacZ expression experiments. Mice were housed in an air-conditioned room, maintained at about 25 °C and 50% humidity. Standard commercial pellet diet and water were available ad libitum. The day at which vaginal plug was observed was designated as E0.5 at noon.

### Tissue processing

Embryo heads were dissected and fixed in 4% paraformaldehyde (PFA) in phosphate-buffered saline (PBS) for 12 h at 4 °C. For postnatal assessments, mice were anesthetized by intraperitoneal injection of 4 mg/kg xylazine (Bayer, Shawnee Mission, KS, USA) and 120 mg/kg ketamine-HCl (Daiichi Sankyo, Tokyo, Japan) in 0.9% NaCl, and fixed by cardiac perfusion of 4% PFA in PBS. Subsequently, inner ears were dissected from temporal bones and decalcified in 0.5 M EDTA (Wako, Osaka, Japan) for 3 days at 25 °C. Cochleae were embedded in OCT (Sakura Finetek Japan, Tokyo, Japan) and serially sectioned at 12 μm thickness by a cryostat.

### β-Galactosidase staining

TSK expression was investigated throughout the developmental stages of the inner ear by staining for LacZ in mice heterozygous for LacZ insertion in TSK. In particular, the inner ear was examined at embryonic stages E9.5, E11.5, E13.5, and E15.5, as well as at P0, P10, and P30 (each stage, *n* = 5). Slides were dried for 30 min at 25 °C, stained with 400 mg/mL X-gal (Genlantis, San Diego, CA, USA) for 18 h at 37 °C, and imaged at 1360 × 1024 pixels using a BZ-9000 microscope (Keyence, Osaka, Japan) using uniform photographic exposure parameters.

### Immunostaining

Sections were blocked with 10% (v/v) normal goat serum in PBS containing 0.1% Triton X-100 (IBI Scientific, Peosta, IA, USA) for 10 min at 25 °C and labeled using rabbit antibody against acetylated α-tubulin (Tuj1; 1:500, COVANCE, Princeton, NJ, USA) and mouse antibody against Sox2 (1:200, Cell Signaling, CA, USA) in a humidified chamber for 1 h at 25 °C. Subsequently, sections were washed and incubated with goat anti-mouse or anti-rabbit IgG conjugated to Alexa 594 (1:500, Thermo Fisher Scientific, Rockford, IL, USA) for 1 h at 25 °C. After washing with PBS, tissue sections were counterstained with Hoechst 33342 (Molecular Probes, OR, USA), mounted, covered with coverslips, and imaged at 1360 × 1024 pixels by using a BZ-9000 fluorescence microscope (Keyence) with uniform photographic exposure parameters. Sections representative of at least five mice were presented.

### Enumeration of hair cells

HCs were counted as described previously [33]. In brief, cochleae were removed from temporal bones at P0, P6, and P30 and fixed for 18 h at 4 °C. Bony capsules and lateral walls were then removed. After blocking with 0.3% Triton X-100 in PBS for 10 min, the organ of Corti (OC) was labeled using rabbit antibody against Myo7a (1:500, Thermo Scientific Pierce, PA1–936) and goat anti-mouse or anti-rabbit IgG conjugated to Alexa 488 (1:500, Thermo Fisher Scientific). Tissue sections were counterstained with Hoechst 33342 (Molecular Probes), mounted, and covered with coverslips for P30 cochleae, while the OC was stained by Texas Red-X phalloidin (Molecular Probes) for 30 min for P0, P6, and P30 cochleae (P0, each *n* = 2; P6, each *n* = 2; P30, each *n* = 5). Between steps, tissue sections were washed with PBS thrice for 5 min each. Surface images of the OCs were captured, and the inner and outer HCs were counted after Myo7a staining over 300 μm^2^ of tissue. The viability of HCs was also assessed (CONT and TSK-KO, each *n* = 5 at P30).

### Enumeration of the spiral ganglion cells

SGCs were counted as described previously [34]. Briefly, after immunostaining for Tuj1 and counterstaining with Hoechst, we delineated the Rosenthal’s canal as the area that contains clusters of doubly stained cells and extends laterally to the modiolus. SGCs in the Rosenthal’s canal, which were positive for both Tuj1 and Hoechst stain, were then marked and counted visually using ImageJ (NIH, Bethesda, MD, USA) at the apical, middle, and basal turns using three randomly selected sections per cochlea (CONT and TSK-KO, each *n* = 5). A second researcher reviewed the results for accuracy and calculated the average.

### Measurement of the thickness of the stria vascularis

The average thickness of the stria vascularis was measured by analyzing images of the sections containing the midmodiolar region by using ImageJ (NIH) (CONT and TSK-KO, each *n* = 3).

### In situ hybridization

Cochleae collected at E13.5, E15.5, and P0 were examined by in situ hybridization according to Moorman et al. [35], by using antisense riboprobes for BMP4 and Sox2 that were labeled with digoxigenin using DIG RNA Labeling Kit (Roche, Indianapolis, IN, USA). The probes were produced using the corresponding DNA constructs. Detailed protocols are available upon request. A minimum of five samples were prepared for each time point (CONT and TSK-KO, each *n* = 5).

### Scanning electron microscopy

Each mouse was anesthetized as described above and briefly perfused with 0.9% NaCl, followed by 20 mL of 2.5% glutaraldehyde and 2 mM CaCl_2_ in 0.1 M sodium cacodylate (pH 7.4), through the ascending aorta. The inner ear was removed by dissection. The top of the cochlea was punctured using a fine-tipped pair of forceps, and semicircular canals were sliced open. The inner ear was then gently flushed through these openings using 0.3 mL of 2.5% glutaraldehyde and subsequently fixed in the same solution for 18 h at 4 °C. The membranous labyrinth containing the cochlea was removed by dissection. Cochlear specimens were prepared by removing the stria vascularis, Reissner’s membrane, and tectorial membrane. The cochlear spiral was sectioned into basal, middle, and apical segments and stained with 1% OsO_4_ thrice for 1 h, based on the technique by Hunter-Duvar [36]. Samples were incubated with saturated thiocarbohydrazide for 20 min after the first and second OsO_4_ treatments. Specimens were then dehydrated in a graded series of ethanol and critical-point-dried using liquid CO_2_ as transitional fluid. Uncoated specimens were mounted on a Hitachi specimen stub, using silver electroconductive paint, and imaged using a Hitachi S-4800 field-emission scanning electron microscope operated at 5 kV. Five mice from each group (CONT and TSK-KO) were studied.

### Quantification of stereocilia

Stereociliary dimensions were measured by using ImageJ (NIH) as described previously [37]. Ten inner HCs per mouse were imaged at 20,000× magnification at basal, middle, and apical turns to measure the average length of the four tallest stereocilia in each HC (CONT and TSK-KO, each *n* = 5).

### Laser microdissection

At E13.5, E15.5, and P0, whole heads were dissected and incubated in 4% PFA for 1 h at 25 °C. Samples were then embedded in OCT, sectioned at 10 μm in the plane of the long axis of the cochlear modiolus, mounted on uncharged slides (Leica Microsystems, Wetzlar, Germany), and dried at 25 °C. Slides were incubated in 95% acetone at − 20 °C and dried at 25 °C immediately before laser microdissection using an LMD7 system (Leica Microsystems), as described by Pagedar et al. [38]. Cell samples were obtained from cochlear epithelium or SGCs. Each slide contained multiple adjacent sections, and all cells in each category were pooled from individual slides onto a single cap (CONT and TSK-KO, each *n* = 5).

### Quantitative reverse-transcription polymerase chain reaction

Using a microRNA extraction kit (QIAGEN, Valencia, CA, USA), total RNA was extracted from each sample obtained by laser microdissection, quantified using a GeneQuant100 (GE Healthcare, Amersham, UK), and diluted as needed to achieve uniform concentrations. cDNA was then synthesized using a One-Step PrimeScript RT-PCR Kit (Takara Bio, Otsu, Japan) according to the manufacturer’s instructions, using primers for *BMP4*, *Sox2*, and *GAPDH* (Applied Bionics, Foster City, CA, USA). Targets were amplified using a Takara Dice TP960 over 40 cycles of denaturation at 95 °C for 15 s and annealing at 60 °C for 1 min. Relative gene expression was calculated by generating a standard curve and normalized to *GAPDH* signal (CONT and TSK-KO, each *n* = 7).

### Auditory function

Mice were anesthetized at P30 as described above, and electrodes were placed beneath the pinna of the test ear and at the vertex just below the surface of the skin. The ground electrode was placed under the contralateral ear. Auditory thresholds were measured at 4, 12, and 20 kHz by recording the ABR (15 ms duration, 1 ms rise/fall time, and tone burst) on a System 3 (Tucker-Davis Technologies, Alachua, FL, USA). For each recording, 1024 sweeps were averaged. Stimulus levels near the threshold were changed in 10-dB steps, and the threshold was defined as the lowest level at which waves in the ABR were clearly detectable by visual inspection (CONT and TSK-KO, each *n* = 5).

### Otoacoustic emissions

Five mice from each group were anesthetized as described above, and pinnae were removed. An ER10B+ probe microphone/speaker system with two speaker ports (Etymotic Research, Inc., Elk Grove Village, IL, USA) was fitted tightly into the ear canal and linked to two closed-field EC-1 speakers (Tucker-Davis Technologies). Two primary tones were generated (1 s duration with 20 ms rise/fall cosine ramp; f 2/f 1 = 1.22, f 2 varied at a one-fourth octave step from 4 to 29 kHz) and routed separately to the two EC-1 speakers at SPL1 = 75 dB and SPL2 = 65 dB. The SPL was calibrated in a 0.1-mL coupler [39] using a Brüel and Kjær 1/4″ pressure field microphone (model 4938), which has a flat frequency response from 4 Hz to 70 kHz. The calibration was conducted for primary tones and all the components of the DPOAE. The DPOAE response from the ER10B+ microphone was amplified by 20 dB and digitized at 150 kHz using a PCI-MIO-16E-1 A/D converter (National Instruments, Austin, TX, USA). Data were acquired and analyzed using customized software written in Matlab (The Mathworks, Natick, MA, USA). Recordings were repeated 10 times at 20-s intervals and averaged as a function of time. The noise was estimated by averaging three adjacent frequency bins that were above and below the DPOAE frequency [40].

### Endocochlear potentials

Endocochlear potentials were recorded at P30 under general anesthesia [41]. The cochlea was firstly exposed ventrally. The bone over the spiral ligament was thinned, and a small opening was generated with a pick to access the endolymphatic compartment (scala media) of the basal turn. A heat-pulled micropipette electrode filled with 150 mM KCl was inserted into this compartment until a stable potential was recorded, at which point potentials no longer depended on the electrode depth. The signal was amplified through an MEZ-7200 amplifier (Nihonkoden, Tokyo, Japan), and direct current potentials were recorded using a USB-6216 A/D converter (National Instruments) (CONT and TSK-KO, each *n* = 3).

### Contextual and cued fear-conditioning test

To assess fear-associated learning and memory, each mouse was placed in a test chamber (33 × 25 × 28 cm) with a stainless-steel grid floor (0.2 cm diameter, spaced 0.5 cm apart; O’HARA & CO., Tokyo, Japan) in a sound-attenuating chamber to control environmental conditions and allowed to explore for 2 min [42]. Subsequently, a conditioned stimulus (CS; 55 dB white noise) was presented for 30 s, followed by a mild foot shock (2 s, 0.3 mA), which served as the unconditioned stimulus (UCS). Two more CS–UCS pairs were presented at 2-min interstimulus intervals. Context test was conducted 1 day after conditioning in the same chamber for 300 s on each mouse. A cued test with an altered context was then conducted in a triangular chamber (33 × 29 × 32 cm) made of white opaque plastic, placed in a different room. Tone stimulus for the cued test (55 dB white noise) was applied for 180 s. In each test, freezing percentage was calculated automatically using the ImageFZ software based on the public domain Image J program and developed and modified by Tsuyoshi Miyakawa (available through O’HARA & CO.) (CONT: *n* = 15, TSK-KO: *n* = 17).

### Experimental design and statistical analyses

Data are reported as mean ± s.d. Two groups were compared using Mann–Whitney *U* test. For comparisons of more than two groups, one-way or two-way repeated-measures ANOVA was performed, followed by Bonferroni’s post-hoc test of pairwise group differences. Statistical calculations were performed using Microsoft Excel (Redmond, WA, USA). Results with a *p* value < 0.05 were considered statistically significant.

## Supplementary information

**Additional file 1: **Behavioral and fear-conditioning tests in the CONT and TSK-KO mice. Freezing responses between the CONT and TSK-KO mice in the conditioning phase (auditory stimulus and foot shock, 2-min interstimulus interval) or contextual testing (1 day after conditioning for 300 s) were not significantly different (conditioning phase, *p* = 0.94; contextual testing, *p* = 0.75). Levels of freezing in the TSK-KO mice significantly decreased during the auditory cue in an altered context (auditory stimulus for 180 s) compared with that in the CONT mice (*p* = 0.02). CONT mice: *n* = 15, TSK-KO mice: *n* = 17. CS: conditioned stimulus, UCS: unconditioned stimulus, N.S.: not significant.

**Additional file 2.** Enumeration of hair cells in surface preparations of cochleae. Stereocilia of inner hair cells at P0 and P6 were likely to be shortened in the surface preparation. IHC: inner hair cell, OHC: outer hair cell.

**Additional file 3.** Schematic drawing of TSK function in the cochlea. (A) TSK function in cochlea epithelium from E13.5 to P30. (B) TSK function in the spiral ganglion from E18.5 to P30. WT: wild-type.

## Data Availability

The datasets used and/or analyzed during the current study are available from the corresponding author on reasonable request.

## Abbreviations

ABR: Auditory brainstem response
CS: Conditioned stimulus
DPOAE: Distortion product of otoacoustic emission
EP: Endocochlear potential
HC: Hair cell
OC: Organ of corti
PBS: Phosphate-buffered saline
PFA: Paraformaldehyde
SGC: Spiral ganglion cell
TSK: Tsukushi
UCS: Unconditioned stimulus
