# Supplementary material for: Tsukushi is essential for the development of the inner ear
Source: Mol Brain. 2020 Mar 3;13:29. doi: 10.1186/s13041-020-00570-z (PMC7053050; doi:10.1186/s13041-020-00570-z)
Supplement: Supplementary file 1 — Additional file 1: Behavioral and fear-conditioning tests in the CONT and TSK-KO mice. Freezing responses between the CONT and TSK-KO mice in the conditioning phase (auditory stimulus and foot shock, 2-min interstimulus interval) or contextual testing (1 day after conditioning for 300 s) were not significantly different (conditioning phase, p = 0.94; contextual testing, p = 0.75). Levels of freezing in the TSK-KO mice significantly decreased during the auditory cue in an altered context (auditory stimulus for 180 s) compared with that in the CONT mice (p = 0.02). CONT mice: n = 15, TSK-KO mice: n = 17. CS: conditioned stimulus, UCS: unconditioned stimulus, N.S.: not significant. [file 13041_2020_570_MOESM1_ESM.pdf]

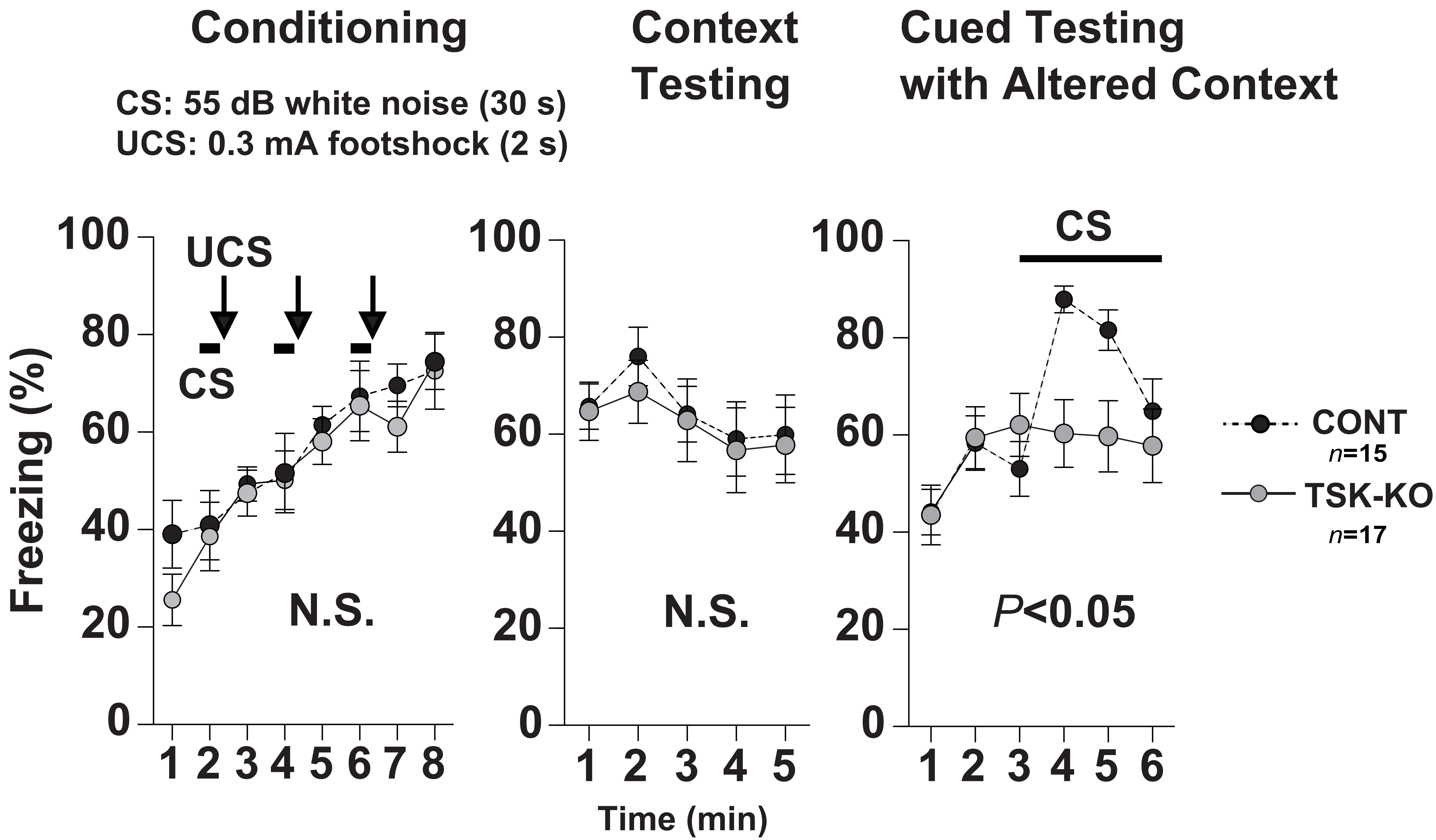
Conditioning
Context Testing
Cued Testing with Altered Context
CS: 55 dB white noise (30 s)
UCS: 0.3 mA footshock (2 s)
Freezing (%)
UCS
CS
N.S.
N.S.
CS
P<0.05
Time (min)
CONT
n=15
TSK-KO
n=17
