# Supplementary figures and images for: Tsukushi is essential for the development of the inner ear
Source: Mol Brain. 2020 Mar 3;13:29. doi: 10.1186/s13041-020-00570-z (PMC7053050; doi:10.1186/s13041-020-00570-z)

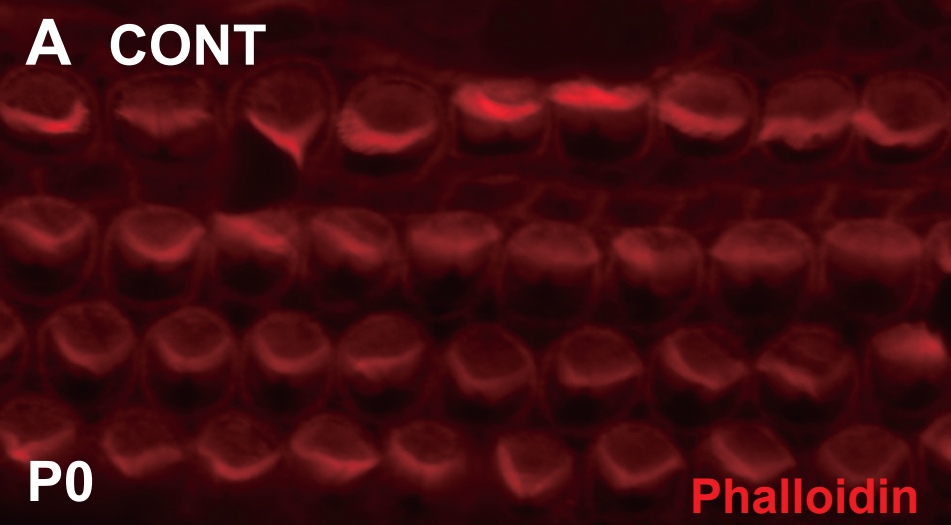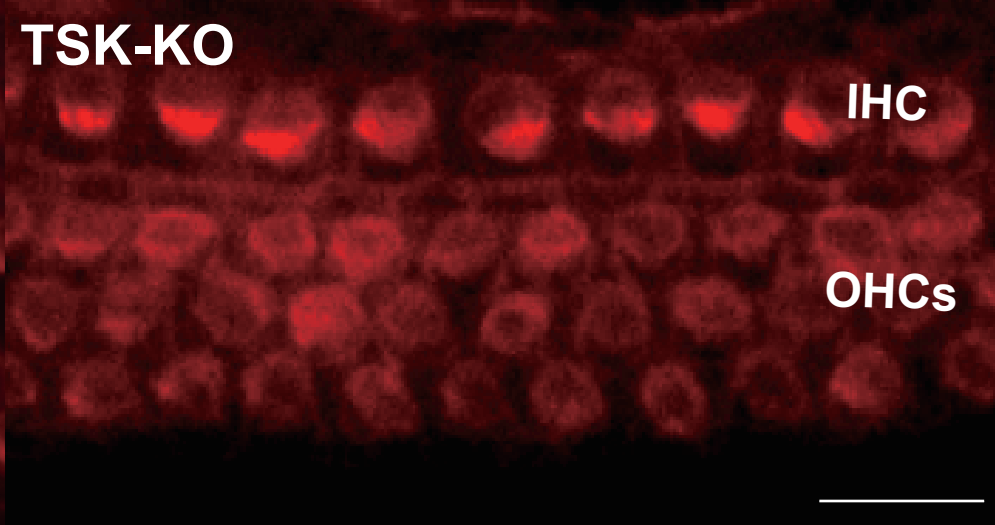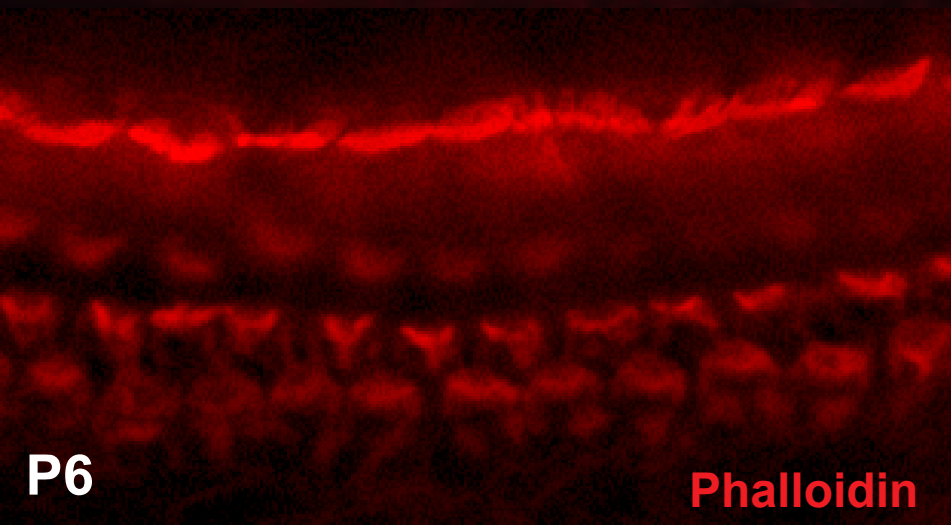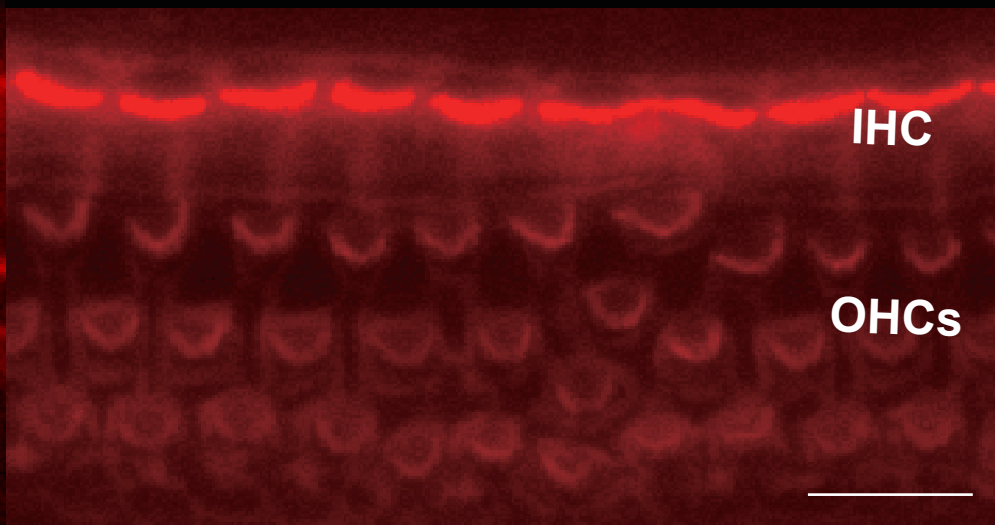

Supplement: Supplementary file 2 — Additional file 2. Enumeration of hair cells in surface preparations of cochleae. Stereocilia of inner hair cells at P0 and P6 were likely to be shortened in the surface preparation. IHC: inner hair cell, OHC: outer hair cell. [file 13041_2020_570_MOESM2_ESM.pdf]

A

## Cochlea epithelium

E13.5-E15.5

E18.5-P30

WT

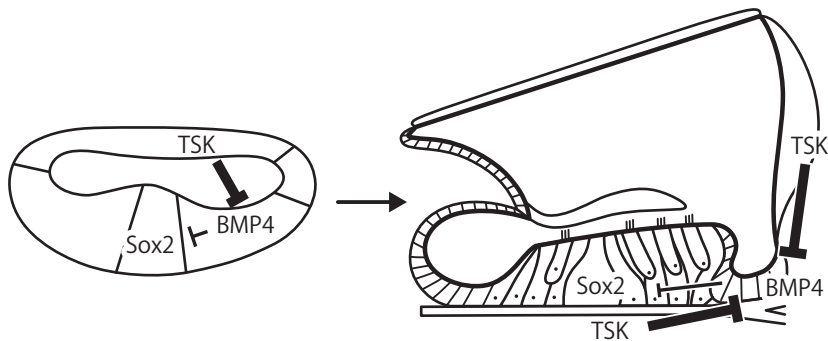

TSK-KO

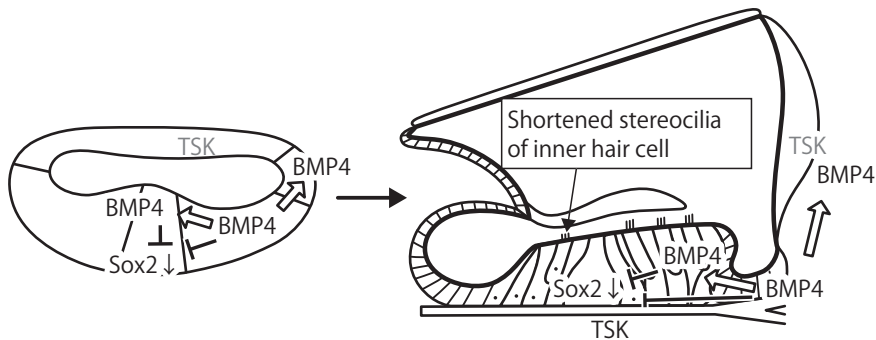

B

Spiral ganglion cells  
in basal turn

E18.5-P30

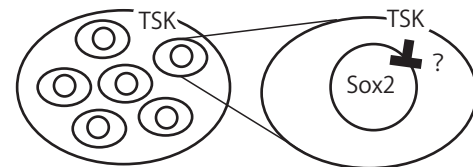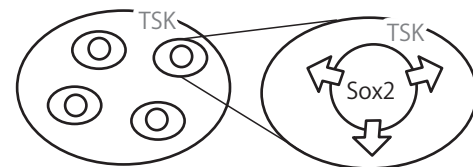

Supplement: Supplementary file 3 — Additional file 3. Schematic drawing of TSK function in the cochlea. (A) TSK function in cochlea epithelium from E13.5 to P30. (B) TSK function in the spiral ganglion from E18.5 to P30. WT: wild-type. [file 13041_2020_570_MOESM3_ESM.pdf]
